# Efficacy and Safety of Second‐Line Immune Checkpoint Inhibitor Rechallenge in Advanced or Metastatic Esophageal Squamous Cell Carcinoma: A Retrospective Study

**DOI:** 10.1111/1759-7714.70131

**Published:** 2025-07-09

**Authors:** Wensi Zhao, Nan Zhao, Dedong Cao

**Affiliations:** ^1^ Department of Oncology Renmin Hospital of Wuhan University Wuhan China

**Keywords:** esophageal squamous cell carcinoma, immune checkpoint inhibitor, overall survival, rechallenge, second‐line setting

## Abstract

**Background:**

Immune checkpoint inhibitor (ICI) has reshaped the treatment landscape of esophageal squamous cell carcinoma (ESCC). But most patients end up with disease progression and/or therapeutic intolerance. The subsequent ICI rechallenge raises some discussions.

**Methods:**

A retrospective study was conducted to assess the efficacy and safety of reintroduction of ICI in patients with advanced or metastatic ESCC after first‐line ICI failure. Outcomes included median overall survival (OS), progression‐free survival (PFS), objective response rate (ORR), disease control rate (DCR) and safety. Subgroup analysis and prognostic analysis were also performed.

**Results:**

A total of 1320 patients were screened and 138 were enrolled: 109 received second‐line ICI‐based therapies, and 29 received non‐ICI therapies. As of data cutoff on November 30, 2024, patients with ICI rechallenge, compared with non‐ICI rechallenge, achieved an improved second‐line OS (10.4 vs. 5.8 months; HR = 0.53, 95% CI: 0.33–0.84; *p* = 0.006) and showed a favorable PFS trend (5.0 vs. 3.0 months; HR = 0.75, 95% CI: 0.48–1.17; *p* = 0.202). The 6‐month PFS rate was 42.9% versus 22.3%, and the 12‐month OS rate was 41.5% versus 23.2%, respectively. The ORR was 30.3% versus13.8% and the DCR was 79.8% versus 58.6%, respectively. ICI combined with chemoradiotherapy was the most popular option for subsequent ICI rechallenge, with an OS of 11.2 months. Treatment‐related adverse events of grade ≥ 3 occurred in 47 (43.1%) and 11 (37.9%) patients in the two groups.

**Conclusion:**

Second‐line ICI rechallenge provided OS benefits in advanced or metastatic ESCC, with manageable safety. Further prospective study is warranted.

## Graphical abstract

Zhao et al. demonstrate the efficacy, survival and safety of second‐line immune checkpoint inhibitor rechallenge in advanced or metastatic esophageal squamous cell carcinoma patients. This provides a potential therapy option of second‐line treatment for esophageal squamous cell carcinoma patients after first‐line immunotherapy failure.

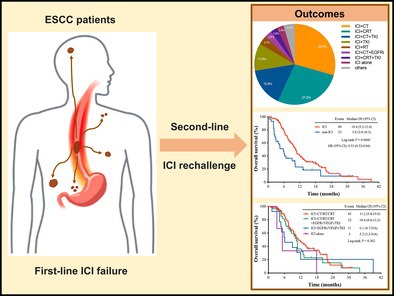

**Abbreviations**
CRT: chemoradiotherapy
DCR: disease control rate
ESCC: esophageal squamous cell carcinoma
ICI: immune checkpoint inhibitor
ORR: objective response rate
OS: overall survival
PFS: progression‐free survival
PSM: propensity score matching
RECIST: response evaluation criteria in solid tumors
TKI: tyrosine kinase inhibitor
TRAEs: treatment‐related adverse events

## 1 Introduction

With an estimated 510 716 new cases and 445 129 cancer deaths worldwide, esophageal cancer (EC) is the eleventh and seventh most common leading cause of cancer morbidity and mortality in 2022 [1]. Due to the unique geographical environment, dietary habits, and genetic factors, China has always been one of the countries with traditionally high incidence and poor prognosis of EC, especially in medium human development index regions. According to the latest report from the National Cancer Center of China in 2022, there are nearly 224 000 new cases of EC and 187 500 deaths in China, 90% of which are esophageal squamous cell carcinoma (ESCC) [2].

Due to the atypical clinical symptoms and the inadequacy of standardized endoscopic screening, most patients with EC are initially diagnosed with advanced disease. Compared with traditional chemotherapy, mounting evidence has confirmed the efficacy and safety of immune checkpoint inhibitor (ICI) in the first line (1L) treatment of locally advanced/recurrent and metastatic ESCC, with a median overall survival (OS) of up to 17.2 months and progression‐free survival (PFS) of 7.2 months [3, 4, 5, 6, 7, 8, 9, 10, 11]. Immunochemotherapy has become the new standard 1L treatment for advanced or metastatic ESCC. However, disease progression and/or treatment intolerance may still occur over time.

For the second‐line (2L) therapy of recurrent or metastatic ESCC, irinotecan and docetaxel are the most commonly used chemotherapy agents, with a median PFS and OS of only 3 and 6 months, respectively, which need to be further improved [12, 13, 14]. Compared with chemotherapy, ICI showed effective anti‐tumor activity and manageable safety profiles at the 2L setting in ESCC patients [15, 16, 17, 18, 19]. In the KEYNOTE‐181 study, pembrolizumab provided a median OS of 10.3 versus 6.7 months with chemotherapy in patients with PD‐L1 (programmed cell death‐ligand 1) combined positive score (CPS) of ≥ 10^17^. The ATTRACTION‐3 study showed an improvement of 2.5 months in OS (10.9 vs. 8.4 months) with nivolumab compared to chemotherapy [16]. In the ESCORT trial, camrelizumab provided a 2L OS of 8.3 vs. 6.2 months with chemotherapy [15]. However, the response to 2L ICI rechallenge therapy after the failure of 1L ICI‐based treatment in ESCC patients has not been reported.

Recently, increasing evidence supported the ICI rechallenge for non‐small‐cell lung cancer (NSCLC), melanoma, hepatocellular carcinoma (HCC), head and neck squamous cell carcinoma (HNSCC) and renal cell cancer (RCC) [20, 21, 22, 23, 24]. For ESCC, in fact, ICI rechallenge is also very common in our clinic practice due to the long‐tail effect of ICI. However, the relevant research is still relatively limited. In addition, more data are needed to address the specific treatment timing, benefit population, combination strategy, and safety management of ICI rechallenge [25]. Therefore, we did the study to compare the efficacy and safety of ICI rechallenge and non‐ICI rechallenge in patients with advanced or metastatic ESCC after 1L ICI failure, aiming to provide more clinical evidence for the 2L treatment of ESCC.

## 2 Methods

### 2.1 Study Design

This retrospective observational study was conducted at the Renmin Hospital of Wuhan University, China. The data of locally advanced/recurrent or distant metastatic ESCC patients between September 2019 and December 2023 were collected. Patients were selected according to the following inclusion and exclusion criteria. Eligible patients were ≥ 18 years of age, had histologically or cytologically confirmed ESCC, had 1L ICI‐based treatment failure, had received at least one dose of 2L treatment, and had available follow‐up data. Patients with other malignancies (except skin basal cell carcinoma and cervical carcinoma in situ) or with incomplete medical data were excluded.

### 2.2 Outcomes

Patients' clinical and survival data were collected retrospectively, including age, sex, histological type, primary tumor location and stage, type of metastasis, metastatic site, PD‐L1 and Ki‐67 expression, EGFR mutation, and treatment information. We classified the type of recurrence or metastasis into three levels: local recurrence, regional lymph node metastasis, and distant metastasis. Metastatic lymph nodes were counted by station and jointly assessed by the eighth edition UICC/AJCC (Union for International Cancer Control/American Joint Committee on Cancer) staging of cancers of the esophagus and esophagogastric junction. PD‐L1 expression was detected by immunohistochemistry (22C3 pharmDx assay, Agilent, Dako). CPS was calculated by adding the number of viable PD‐L1 positive tumor cells to the number of positive tumor‐infiltrating immune cells, divided by the total number of viable tumor cells, with a maximum score of 100. We set a range of thresholds for CPS (≥ 1 or 10).

Follow‐up was based on the electronic medical records of inpatients or outpatients and/or telephone interviews by investigators until November 30, 2024. Patients who received at least one dose of 2L treatment were included in the efficacy and safety analyses. Tumor responses were evaluated every two treatment cycles according to the Response Evaluation Criteria in Solid Tumors (RECIST) 1.1 and classified as complete response (CR), partial response (PR), stable disease (SD), and progressive disease (PD) until death or the end of the study. Objective response rate (ORR) was determined as the rate of a best response of CR or PR. Disease control rate (DCR) included CR, PR, and SD. OS1 and OS2 were defined as the time since 1L or 2L treatment initiation to death from any cause or date of last follow‐up. PFS1 and PFS2 were defined as the time since 1L or 2L treatment initiation to disease progression or death from any cause or date of last follow‐up. Treatment‐related adverse events (TRAEs) and immune‐mediated adverse events (irAEs) were collected according to the Medical Dictionary for Regulatory Activities v23.0, and the severity was graded according to the National Cancer Institute Common Terminology Criteria for Adverse Events v4.03.

### 2.3 Statistical Analysis

Statistical analyses were undertaken using SPSS v26.0. Categorical variables were summarized by percentages and compared by chi‐squared test or Fisher's exact test, as appropriate. Continuous variables were described by median and interquartile range (IQR). Median follow‐up time was determined using the reverse Kaplan–Meier estimator. Median PFS and OS were plotted using the Kaplan–Meier method. The survival distributions were compared using the Log‐rank test. A Cox proportional hazards regression model was performed to calculate the hazard ratio (HR) and bilateral 95% confidence interval (CI). A multivariate cox regression analysis of OS was used to evaluate the treatment effect after adjusting for important prognostic factors. A forest plot was generated for visual representation of the subgroup analysis. Propensity score matching (PSM) was performed using R v4.2.3 as an exploratory post hoc survival analysis with a 1:1 matching ratio and a caliper of 0.02. Treatment and disease progression patterns, demographic characteristics, TRAEs, and other clinical data were summarized descriptively. A *p*‐value of < 0.05 was considered to be statistically significant.

## 3 Results

### 3.1 Patient Characteristics

**FIGURE 1:**
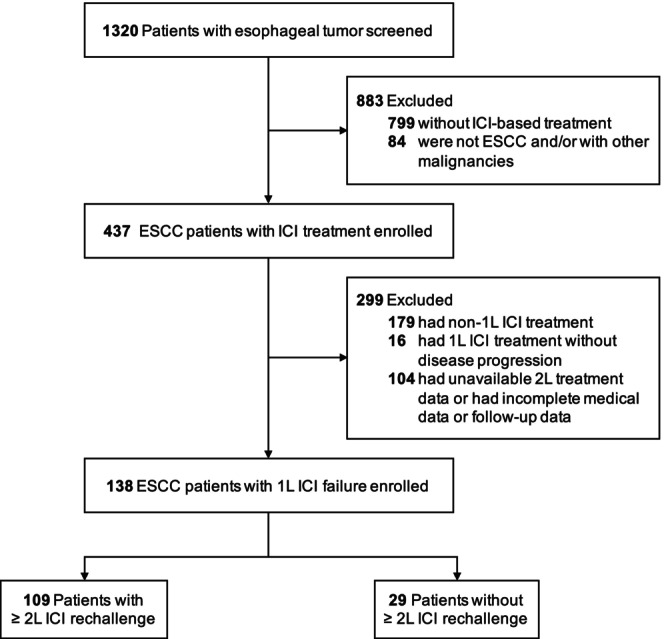
Flowchart of the study. 1L, first‐line; 2L, second‐line; ESCC, esophageal squamous cell carcinoma; ICI, immune checkpoint inhibitor.

In total, 138 patients with recurrent or metastatic ESCC after 1L ICI failure were included in the present study (Figure 1); 109 (79.0%) had 2L ICI rechallenge treatment (ICI cohort) and 29 (21.0%) had 2L non‐ICI rechallenge treatment (non‐ICI cohort). Of the entire cohort, the median age of the patients was 62 years (IQR, 58–68 years), 119 (86.2%) patients were male, 136 (98.6%) had an ECOG PS score of 0–1, 111 (80.5%) had primary tumors located in the middle and lower esophagus, 124 (89.9%) had stage III/IV primary tumors, 116 (84.1%) had local lymph node metastasis, and 80 (58.0%) had distant metastasis. Only about 12% of patients have clearly defined PD‐L1 expression data. Gene mutation data was available for 54 patients (39.1%), and 52 (37.7%) were EGFR mutants. The majority of patients had previously been exposed to chemotherapy (95.7%) and radiotherapy (60.1%). All patients received 1L ICI‐based treatment. Patient baseline characteristics, stratified according to the subsequent ICI rechallenge or not, are presented in Table 1. There was no statistically significant difference in baseline characteristics between the two groups (Table S1).

**TABLE 1:** Patient demographics and baseline characteristics.

Characteristics	Patients, no. (%)			*p*
	ICI cohort (*N* = 109)	Non‐ICI cohort (*N* = 29)	All (*N* = 138)	
Age, median (IQR), year	62 (58–67)	63 (60–70)	62 (58–68)	0.176
≥ 60	68 (62.4)	22 (75.9)	90 (65.2)	
< 60	41 (37.6)	7 (24.1)	48 (34.8)	
Sex				> 0.999
Male	94 (86.2)	25 (86.2)	119 (86.2)	
Female	15 (13.8)	4 (13.8)	19 (13.8)	
ECOG PS score				> 0.999
0–1	107 (98.2)	29 (100.0)	136 (98.6)	
2	2 (1.8)	0 (0.0)	2 (1.4)	
Primary tumor stage				> 0.999
I–II	11 (10.1)	3 (10.3)	14 (10.1)	
III–IV	98 (89.9)	26 (89.7)	124 (89.9)	
Primary tumor location				0.088
Upper esophagus	19 (17.4)	2 (6.9)	21 (15.2)	
Middle esophagus	55 (50.5)	21 (72.4)	76 (55.1)	
Lower esophagus	31 (28.4)	4 (13.8)	35 (25.4)	
Unknown	4 (3.7)	2 (6.9)	6 (4.3)	
Type of metastasis				0.731
Local recurrence^a^	18 (16.5)	6 (20.7)	24 (17.4)	
Regional LN metastasis^b^	94 (86.2)	22 (75.9)	116 (84.1)	
Distant metastasis	64 (58.7)	16 (55.2)	80 (58.0)	
Previous treatment				
Surgery	54 (49.5)	9 (31.0)	63 (45.7)	0.075
Chemotherapy	105 (96.3)	27 (93.1)	132 (95.7)	0.806
Radiotherapy	67 (61.5)	16 (55.2)	83 (60.1)	0.538
Immunotherapy^c^	109 (100.0)	29 (100.0)	138 (100.0)	> 0.999
EGFRi/VEGFi/TKI^d^	21 (19.3)	9 (31.0)	30 (21.7)	0.172
PD‐L1^e^				0.406
CPS < 1	2 (1.8)	1 (3.4)	3 (2.2)	
1 ≤ CPS < 10	5 (4.6)	2 (6.9)	7 (5.1)	
CPS ≥ 10	7 (6.4)	0 (0.0)	7 (5.1)	
Unknown	95 (87.2)	26 (89.7)	121 (87.7)	
EGFR status				0.625
WT	2 (1.8)	0 (0.0)	2 (1.4)	
MT	43 (39.5)	9 (31.0)	52 (37.7)	
Unknown	64 (58.7)	20 (69.0)	84 (60.9)	
Ki67% expression				0.611
≤ 30	6 (5.5)	3 (10.3)	9 (6.5)	
> 30	27 (24.8)	6 (20.7)	33 (23.9)	
Unknown	76 (69.7)	20 (69.0)	96 (69.6)	

Abbreviations: CPS, combined positive score; ECOG PS, Eastern Cooperative Oncology Group performance status; EGFR (i), epidermal growth factor receptor (inhibitor); ICI, immune checkpoint inhibitor; IQR, interquartile range; LN, lymph node; MT, mutated type; PD‐L1, programmed cell death‐ligand 1; TKI, tyrosine kinase inhibitor; VEGFi, vascular endothelial growth factor inhibitor; WT, wild type.

^a^ Local recurrence: esophageal or anastomotic recurrence.

^b^ All regional LN metastases were assessed by the eighth edition UICC/AJCC staging of cancers of the esophagus and esophagogastric junction. Metastatic LNs were counted by station.

^c^ Immunnotherapy: anti‐PD‐1 monoclonal antibody (pembrolizumab, nivolumab, camrelizumab, toripalimab, sintilimab, tislelizumab, penpulimab), anti‐PD‐L1 monoclonal antibody (atezolizumab, durvalumab, envafolimab, adebrelimab) and anti‐PD‐1/CTLA4 antibody (cadonilimab).

^d^ EGFRi: cetuximab and nimotuzumab; VEGFi: bevacizumab; TKI: apatinib and anlotinib.

^e^ PD‐L1 expression was detected by immunohistochemistry (22C3 pharmDx assay, Agilent, Dako). Combined positive score (CPS) was calculated by adding the number of viable PD‐L1 positive tumor cells to the number of positive tumor‐infiltrating immune cells, divided by the total number of viable tumor cells, with a maximum score of 100. We set a range of thresholds for CPS (≥ 1 or 10).

### 3.2 Treatment Regimens

**FIGURE 2:**
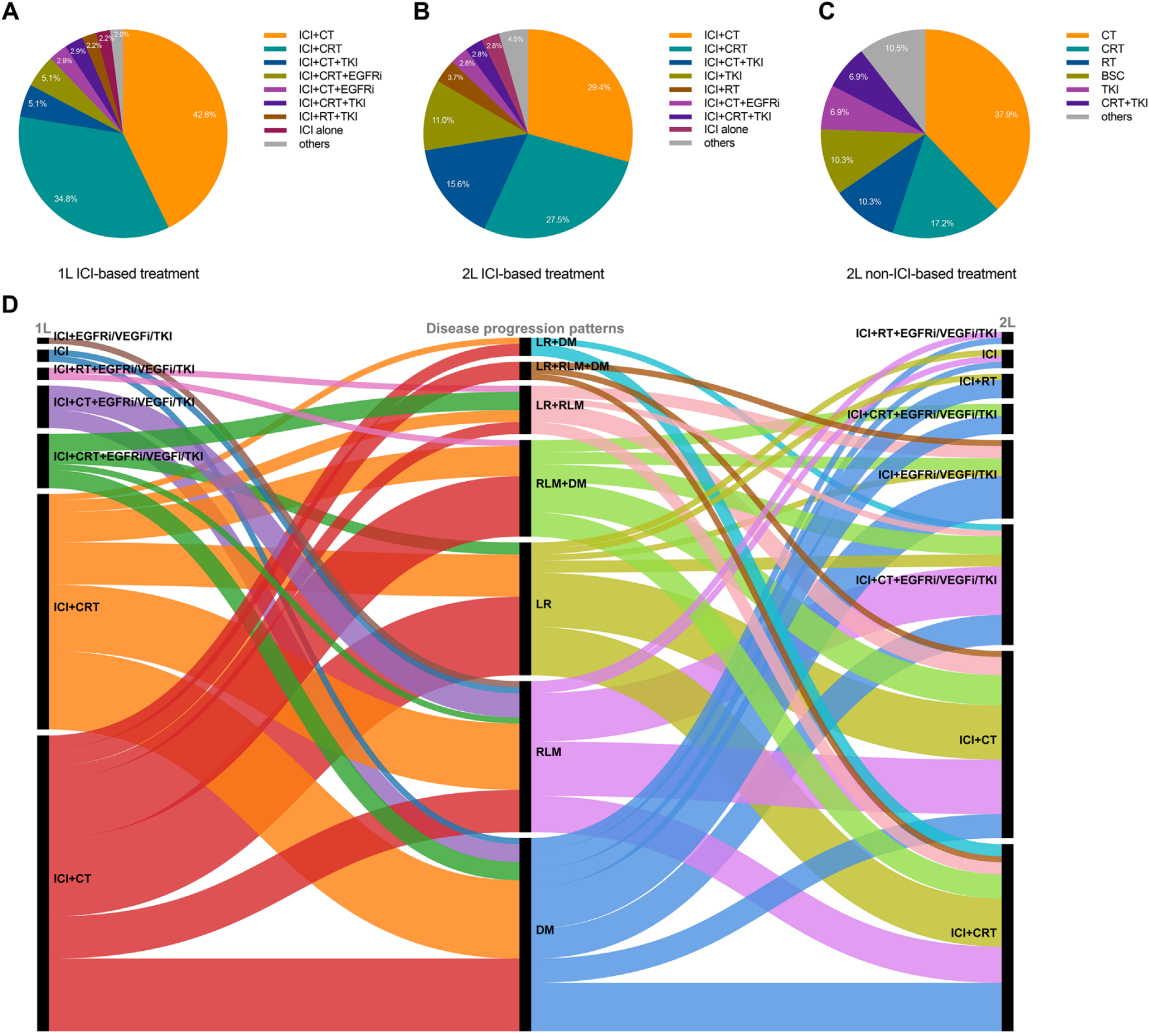
Treatment and disease progression patterns and proportions. (A) Treatment regimens in patients at 1L setting (*N* = 138). (B) Treatment regimens in patients of 2L ICI rechallenge group (*N* = 109). (C) Treatment regimens in patients of 2L non‐ICI group (*N* = 29). (D) Summary of treatment sequences and disease progression patterns in patients of ICI rechallenge group (*N* = 109). 1L, first‐line; 2L, second‐line; BSC, best supportive care; CRT, chemoradiotherapy; CT, chemotherapy; DM, distant metastasis; EGFRi, epidermal growth factor receptor inhibitor (cetuximab and nimotuzumab); ICI, immune checkpoint inhibitor; LR, local recurrence; RLM, regional lymph node metastasis; RT, radiotherapy; TKI, tyrosine kinase inhibitor (apatinib and anlotinib); VEGFi, vascular endothelial growth factor inhibitor (bevacizumab).

The most commonly used regimens in 1L therapy were ICI combined with chemotherapy (42.8%) and ICI combined with chemoradiotherapy (CRT) (34.8%) (Figure 2A). The 2L therapy is still dominated by CRT. In the non‐ICI rechallenge group, chemotherapy and CRT accounted for 37.9% and 17.2%, respectively (Figure 2C). In the ICI rechallenge group, ICI in combination with chemotherapy and CRT accounted for 29.4% and 27.5%, respectively (Figure 2B). Besides, tyrosine kinase inhibitor (TKI) has found its way into the 2L treatment for ESCC—specifically, ICI‐TKI doublet accounted for 11.0%, ICI‐TKI‐chemotherapy triplet has a weight of 15.6%, and ICI‐TKI‐CRT accounted for 2.5%. Broadly, the proportion of patients receiving ICI combined with TKI (1L: 10.2%; 2L: 29.4%) increased gradually with each advancing line of therapy (Figure 2). Less than 3% of patients received ICI monotherapy, and ICI‐based combinations remained the mainstay options for advanced ESCC. Among 109 patients in the ICI rechallenge group, 73 (66.9%) developed disease progression with local lymph node metastasis and/or distant metastasis, and 36 (33.0%) developed local recurrence (Figure 2D). For 49 patients who had previously received 1L ICI combined with chemotherapy, the majority (42.9%, 21/49) received ICI plus CRT at the 2L setting, followed by ICI plus chemotherapy (18.4%, 9/49) and ICI plus target therapy, such as anti‐EGFR/VEGF monoclonal antibody or TKI (14.3%, 7/49) (Figure 2D). For 39 patients who had received ICI plus CRT as 1L treatment, the main options of their 2L treatment included ICI plus chemotherapy (48.7%, 19/39), ICI plus CRT (17.9%, 7/39) and ICI plus chemotherapy and target therapy (12.8%, 5/39) (Figure 2D).

### 3.3 Tumor Response and Survival Prognosis

The ORR of 2L treatment of the two cohorts was 30.3% versus 13.8%; the DCR was 79.8% versus 58.6%, respectively (Table 2). Thirty‐three (30.3%) cases of confirmed PR were reported in the ICI rechallenge cohort. Four (13.8%) cases of PR in the non‐ICI rechallenge cohort were reported. The 3‐month and 6‐month PFS2 rates were 80.5% versus 48.3% and 42.9% versus 22.3%, respectively. And the 12‐month and 24‐month OS2 rates were 41.5% versus 23.2% and 13.3% versus 9.3%, respectively (Table 2).

**TABLE 2:** Tumor response.

	ICI cohort (*N* = 109)	Non‐ICI cohort (*N* = 29)
Best overall response, no. (%)		
CR	0	0
PR	33 (30.3)	4 (13.8)
SD	54 (49.5)	13 (44.8)
PD	21 (19.3)	10 (34.5)
NE	1 (0.9)	2 (6.9)
ORR, % (95% CI)	30.3 (22.0–39.9)	13.8 (4.5–32.6)
DCR, % (95% CI)	79.8 (70.8–86.7)	58.6 (39.1–75.9)
PFS rate, % (95% CI)		
3 months	80.5 (73.4–88.4)	48.3 (33.1–70.4)
6 months	42.9 (34.4–53.5)	22.3 (11.1–44.8)
12 months	12.7 (7.6–21.3)	14.9 (6.0–36.5)
OS rate, % (95% CI)		
6 months	78.8 (71.4–87.1)	43.8 (28.8–66.6)
12 months	41.5 (32.8–52.6)	23.2 (11.3–47.4)
24 months	13.3 (6.8–25.7)	9.3 (1.8–46.9)

Abbreviations: CI, confidence interval; CR, complete response; CT, chemotherapy; DCR, disease control rate; ICI, immune checkpoint inhibitor; NE, not evaluable; OR, odds ratio; ORR, objective response rate; PD, progressive disease; PR, partial response; SD, stable disease.

**FIGURE 3:**
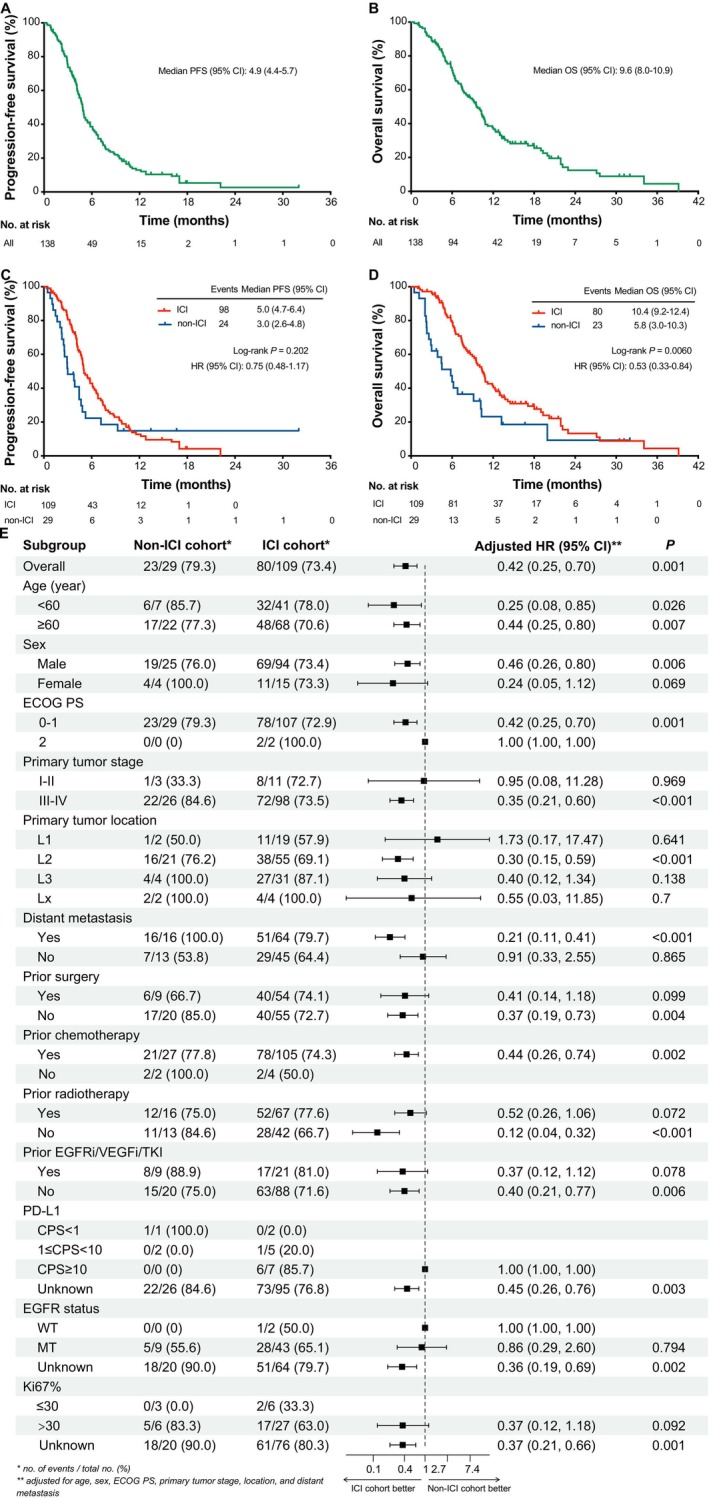
Survival curves and subgroup analysis of second‐line treatment. (A, C) Kaplan–Meier estimates of PFS. (B, D) Kaplan–Meier estimates of OS. (E) Forest plot analyses of OS in subgroups. CI, confidence interval; CPS, combined positive score; ECOG PS, Eastern Cooperative Oncology Group performance status; EGFRi, epidermal growth factor receptor inhibitor; HR, hazard ratio; ICI, immune checkpoint inhibitor; MT, mutated type; OS, overall survival; PFS, progression‐free survival; PD‐L1, programmed cell death‐ligand 1; TKI, tyrosine kinase inhibitor; VEGFi, vascular endothelial growth factor inhibitor; WT, wild type.

**FIGURE 4:**
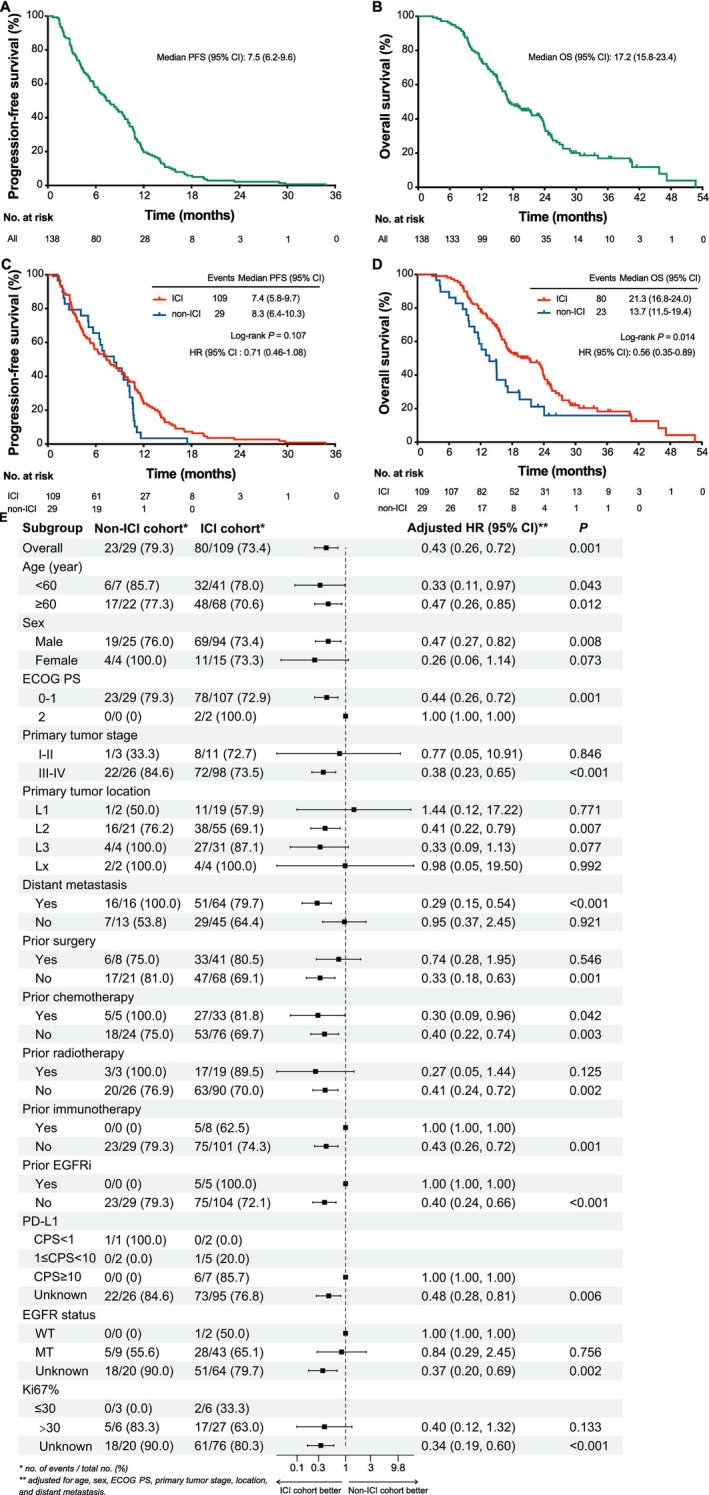
Survival curves and subgroup analysis of first‐line ICI‐based treatment. (A, C) Kaplan–Meier estimates of PFS. (B, D) Kaplan–Meier estimates of OS. (E) Forest plot analyses of OS1 in subgroups. CI, confidence interval; CPS, combined positive score; ECOG PS, Eastern Cooperative Oncology Group performance status; EGFRi, epidermal growth factor receptor inhibitor; HR, hazard ratio; ICI, immune checkpoint inhibitor; MT, mutated type; OS, overall survival; PFS, progression‐free survival; PD‐L1, programmed cell death‐ligand 1; TKI, tyrosine kinase inhibitor; VEGFi, vascular endothelial growth factor inhibitor; WT, wild type.

**FIGURE 5:**
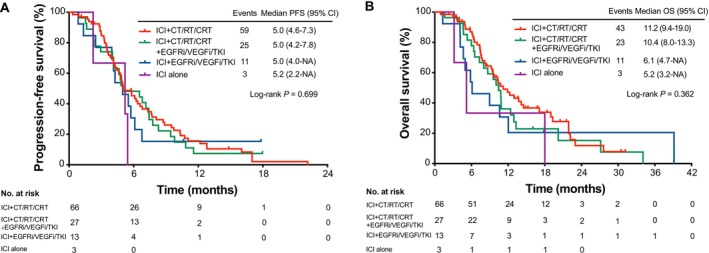
Survival curves of different treatment regimens in second‐line ICI rechallenge. (A) Kaplan–Meier estimates of PFS. (B) Kaplan–Meier estimates of OS. CI, confidence interval; CRT, chemoradiotherapy; CT, chemotherapy; EGFRi, epidermal growth factor receptor inhibitor; HR, hazard ratio; ICI, immune checkpoint inhibitor; NA, not applicable; OS, overall survival; PFS, progression‐free survival; RT, radiotherapy; TKI, tyrosine kinase inhibitor; VEGFi, vascular endothelial growth factor inhibitor.

As of data cutoff on November 30, 2024, with a median follow‐up of 18.4 months, the median PFS2, OS2, PFS1, and OS1 of the whole cohort were 4.9 months (95% CI: 4.4–5.7), 9.6 months (95% CI: 8.0–10.9), 7.5 months (95% CI: 6.2–9.6) and 17.1 months (95% CI: 15.8–23.4), respectively (Figures 3A,B and 4A,B). Patients in the ICI rechallenge cohort achieved a significantly prolonged OS2 (10.4 vs. 5.8 months; HR = 0.53, 95% CI: 0.33–0.84; *p* = 0.006) compared with the non‐ICI rechallenge cohort, and showed a favorable PFS2 trend (5.0 vs. 3.0 months; HR = 0.75, 95% CI: 0.48–1.17; *p* = 0.202) (Figure 3C,D). After PSM, 27 pairs of patients were matched (Table S1). Significantly improved OS2 (10.9 vs. 6.0 months; HR = 0.54; 95% CI: 0.28–1.01; *p* = 0.050) was also exhibited in the ICI rechallenge cohort; the PFS2 (4.9 vs. 3.0 months) displayed a directional improvement (HR = 0.75; 95% CI, 0.42–1.34; *p* = 0.327) (Figure S1A–D). For specific combinations, ICI combined with chemotherapy or radiotherapy or CRT, as the most common options for ICI rechallenge, showed a relatively longest OS2 of 11.2 months (95% CI: 9.4–19.0), although PFS2 differed little (Figure 5A,B). Univariate and multivariate Cox regression analysis showed that EGFR mutation (HR = 0.55, 95% CI: 0.34–0.88; *p* = 0.014) was an independent positive factor associated with OS2 in the ICI rechallenge cohort (Tables S2, S3).

As for the OS1, significant improvement (23.1 vs. 13.7 months; HR = 0.56; 95% CI: 0.35–0.89; *p* = 0.014) was exhibited in the ICI rechallenge cohort as expected (Figure 4D), though the PFS1 of this cohort was shorter than that of the non‐ICI rechallenge cohort (7.4 vs. 8.3 months; HR = 0.71; 95% CI: 0.46–1.08; *p* = 0.107) (Figure 4C). After PSM, the survival benefit of OS1 was also exhibited in the ICI rechallenge cohort (Figure S1E–F). Univariate and multivariate analyses indicated that ECOG PS of 0/1 (HR = 0.17, 95% CI: 0.04–0.74; *p* = 0.018), EGFR mutation (HR = 0.63, 95% CI: 0.40–0.98; *p* = 0.038), PD‐L1 of 1 ≤ CPS < 10 (HR = 0.07, 95% CI: 0.01–0.57; *p* = 0.013), Ki‐67% of ≤ 30 (HR = 0.17, 95% CI: 0.04–0.76; *p* = 0.020) and 2L ICI rechallenge (HR = 0.37, 95% CI: 0.22–0.61; *p* < 0.001) were independent prognostic factors for OS1 (Table S4).

### 3.4 Subgroup Analysis

Some subgroups exhibited a therapeutic effect for OS2 in the forest plot, favoring ICI rechallenge, including patients of all ages, male, ECOG PS of 0–1, Stage III/IV, primary tumor located in the middle esophagus, distant metastasis, a history of prior chemotherapy, and no previous surgery, radiotherapy, or anti‐EGFR/VEGF/TKI target therapy (Figure 3E). Subgroup analyses were also conducted for OS1, and the results were shown in Figure 4E.

### 3.5 Safety

TRAEs of any grade occurred in 107 (98.2%) patients in the ICI rechallenge group versus 28 (96.6%) in the non‐ICI rechallenge group (Table 3). TRAEs of Grade 3 or higher occurred in 47 (43.1%) patients in the ICI group versus 11 (37.9%) in the non‐ICI group; leukopenia (22.0% vs. 20.7%), neutropenia (15.6% vs. 17.2%) and thrombocytopenia (14.7% vs. 10.3%) were the most common. No unexpected safety concerns were reported and no treatment‐related deaths occurred.

**TABLE 3:** Treatment‐related adverse events.

Adverse events, no. (%)	ICI cohort (*N* = 109)		Non‐ICI cohort (*N* = 29)	
	Any grade	Grade ≥ 3	Any grade	Grade ≥ 3
Any	107 (98.2)	47 (43.1)	28 (96.6)	11 (37.9)
White blood cell count decreased	59 (54.1)	24 (22.0)	18 (62.1)	6 (20.7)
Neutrophil count decreased	61 (56.0)	17 (15.6)	15 (51.7)	5 (17.2)
Anemia	64 (58.7)	9 (8.3)	18 (62.1)	3 (10.3)
Platelet count decreased	72 (66.1)	16 (14.7)	14 (48.3)	3 (10.3)
Nausea and/or vomiting	36 (33.0)	2 (1.8)	12 (41.4)	1 (3.4)
Fever	21 (19.3)	5 (4.6)	7 (24.1)	2 (6.9)
Fatigue	34 (31.2)	3 (2.8)	13 (44.8)	1 (3.4)
Appetite decreased	27 (24.8)	0	10 (34.5)	1 (3.4)
Electrolyte disturbance	30 (27.5)	7 (6.4)	10 (34.5)	2 (6.9)
Weight decreased	23 (21.1)	3 (2.8)	8 (27.6)	1 (3.4)
ALT/AST increased	39 (35.8)	8 (7.3)	9 (31.0)	2 (6.9)
Blood creatinine increased	16 (14.7)	0	6 (20.7)	0
Proteinuria	35 (32.1)	6 (5.5)	5 (17.2)	1 (3.4)
Myocardial zymogram increased	18 (16.5)	2 (1.8)	3 (10.3)	0
Pneumonia^a^	30 (27.5)	8 (7.3)	7 (24.1)	2 (6.9)
Hypoalbuminemia	24 (22.0)	3 (2.8)	6 (20.7)	1 (3.4)
Fecal occult blood	17 (15.6)	4 (3.7)	4 (13.8)	1 (3.4)
Hypertension	19 (17.4)	2 (1.8)	3 (10.3)	0
Hyperglycemia	13 (11.9)	2 (1.8)	2 (6.9)	0
Mucositis^b^	29 (26.6)	1 (0.9)	5 (17.2)	0
Rash/pruritus^c^	44 (40.4)	7 (6.4)	5 (17.2)	1 (3.4)
Hypothyroidism/hyperthyroidism	37 (33.9)	3 (2.8)	6 (20.7)	0

*Note:* Events occurring in ≥ 10% of patients in either group are listed.

Abbreviations: ALT, Alanine aminotransferase; AST, Aspartate aminotransferase; ICI, immune checkpoint inhibitor.

^a^ Pneumonia includes lung infection, interstitial pneumonia, radiation‐induced pulmonary injury, and COVID‐19.

^b^ Mucositis includes events of esophagitis, stomatitis, colitis, oral mucositis, and oral inflammation.

^c^ Rash includes erythema, maculopapular, psoriasis, blister, dermatitis bullosa, and reactive cutaneous capillary endothelial proliferation.

As for the irAEs, the incidence of any grade irAEs in the 1L ICI‐based treatment was 44.9% (62 of 138 cases). Rash, pneumonitis, thyroid dysfunction, and abnormal liver function were the most commonly observed irAEs. Grade ≥ 3 irAEs were observed in 13.8% of patients (19 of 138 cases). In the course of 2L ICI rechallenge, the incidence of irAEs of any grade was 39.4% (43 of 109 cases), with grade ≥ 3 irAEs occurring in 18.3% of patients (20 of 109 cases). In general, most of the irAEs were graded 1–2. For grade ≥ 3 irAEs, most of them can be controlled after timely and appropriate symptomatic treatment.

## 4 Discussion

In this study, ICI rechallenge provided significant and clinically meaningful improvements in OS and ORR compared with non‐ICI rechallenge therapy in patients with advanced or metastatic ESCC in the 2L setting. To our knowledge, this is the first study to show a significant survival benefit with ICI reintroduction in the 2L treatment for ESCC after 1L ICI failure.

As is known to all, the results of a series of phase III clinical trials, including KEYNOTE‐590, CheckMate‐648, ESCORT‐1st, ORIENT‐15, JUPITER‐06, and RANTIONALE‐306, have fully established the position of immunochemotherapy as a new standard 1L treatment for advanced or metastatic ESCC, with the median OS1 increasing from 7.0 to 13.0 months of chemotherapy alone to 15.0–17.2 months [3, 4, 5, 6, 7, 8, 9, 10, 11]. Despite the encouraging results, disease progression and drug resistance are inevitable, especially the resistance to checkpoint blockades [26].

Even in melanoma that is most sensitive to ICI treatment, up to 60% of patients have been reported to have inevitably experienced primary resistance. What is even more frustrating is that a subset of patients who initially responded to ICI later developed relapse or disease progression, that is, acquired resistance or secondary resistance. The resistance mechanisms of ICI are complex and vary from patient to patient, mainly including tumor‐intrinsic and tumor‐extrinsic factors, such as JAK1/JAK2 mutations, STK11/LKB1 alterations, PTEN loss‐mediated PI3K activation, immunosuppressive tumor microenvironment, and gut microbiome imbalance, etc. [27, 28, 29, 30, 31]. Strategies in combination with multiple agents with different mechanisms of action have been actively evaluated to prevent and overcome ICI resistance, such as radiotherapy, antiangiogenic therapy, anti‐CTLA‐4/TIGIT antibody, T‐Cell engineering, vaccines, and microbiome‐based therapy [32, 33].

The long‐lasting immunologic memory underpins the durability of immunotherapy, so much so that the reuse of ICI is not uncommon in the clinic. Recently, some clinical studies have shown that the reintroduction of ICI provided an ORR ranged from 0% to 54%, a PFS between 1.5 and 21 months, and an OS ranged from 6.5 months to not reached in patients with melanoma, NSCLC, HNSCC, HCC, RCC, and ESCC [20, 21, 22, 23, 24, 34]. Furthermore, better outcomes were seen in patients with good ECOG PS score, long initial ICI duration, discontinuation of ICI not caused by disease progression, and ICI sequence not switching between anti‐PD‐1 and anti‐PD‐L1. Based on these available data, ICI rechallenge may be a potential strategy for selected patients.

For advanced or metastatic ESCC patients who progressed on 1L ICI treatment, it is worthwhile to further study the ICI rechallenge at 2L setting. Our preliminary results showed that the OS1 (23.1 vs. 13.7 months) and OS2 (10.4 vs. 5.8 months) were significantly improved with 2L ICI rechallenge compared with non‐ICI rechallenge therapy in ESCC patients after 1L ICI failure. Besides, the 2L ICI rechallenge also showed a directional improvement in PFS2 (5.0 vs. 3.0 months), even though the PFS1 (7.4 vs. 8.3 months) was shorter than that of the non‐ICI rechallenge therapy. The higher ORR (30.3% vs. 13.8%), the estimated 12‐month OS2 rate (41.5% vs. 23.2%) and almost doubling 6‐month PFS2 rate (42.9% vs. 22.3%) all indicate the durability of the survival benefit under ICI rechallenge. The estimated OS1 rates at 12, 24, and 36 months in two groups were 77.7% vs. 58.6%, 39.9% vs. 21.2%, and 18.3% vs. 15.9%, respectively, also suggesting a consistent survival benefit brought by ICI rechallenge.

In the present study, we also preliminarily investigated the factors affecting the therapeutic efficacy of ICI in patients with advanced ESCC, both during 1L and upon 2L ICI rechallenge treatment. ICI rechallenge therapy was an independent prognostic factor for OS1, reducing the risk of death by 63%. EGFR mutation is an independent positive factor associated with OS1 and OS2. In fact, anti‐EGFR therapy represented by nimotuzumab has been widely used in advanced ESCC patients in China. More recently, a study has shown that ICI rechallenge in combination with anti‐EGFR agents provided longer PFS (*p* < 0.001) and better DCR (*p* = 0.022) than chemotherapy for recurrent/metastatic nasopharyngeal carcinoma patients who progressed from prior ICI therapy [35]. However, the specific mechanism of EGFR mutation affecting the therapeutic effect of ICI in ESCC remains to be further studied in the laboratory. In addition, we have initially identified some appropriate factors to determine the potential benefit population for ICI rechallenge. Patients of all ages, male, Stage III/IV, with distant metastasis, prior chemotherapy, and no prior surgery, radiotherapy, anti‐EGFR/VEGF or TKI target therapies seem to be able to achieve improvement in OS2 from ICI rechallenge. Of course, this finding needs to be taken with caution, and a prospective randomized study designed with a pre‐specified subgroup analysis is needed for further elucidation.

A study summarized a consensus reached by 147 experts on ICI rechallenge for NSCLC [36]. The results showed that a total of 97.7% of experts agreed to ICI rechallenge; 48.9% voted for rechallenge with the original ICI; 40.3% agreed to rechallenge directly after disease progression; and 88.1% agreed to ICI rechallenge in combination with other agents. For ESCC, increasing studies also have begun to explore the efficacy and safety of ICI rechallenge. A recently published study of 45 advanced ESCC patients receiving two lines of ICI‐based treatments showed that ICI rechallenge, mainly composed of ICI and an angiogenesis inhibitor, provided a PFS2 of 3.2 months and a DCR of 73.3%, with a favorable safety profile compared to front‐line ICI treatment (any grade TRAEs: 40% vs. 64.4%; Grades 3–4 TRAEs: 9.1% vs. 9.1%) [34]. The CAP 02 Re‐challenge study reported a PFS of 4.6 months, an OS of 7.5 months, and grade ≥ 3 TRAEs of 34.7% in patients with advanced ESCC rechallenged with camrelizumab in combination with apatinib [37].

Unlike the combination of ICI and TKI, which is common in HCC or RCC, ICI combined with chemotherapy or CRT ranked first and second among various combinations in the ICI rechallenge group in our study. Chemotherapy or CRT remains the cornerstone for advanced ESCC. The addition of ICI to chemotherapy or CRT may be more beneficial to the survival of ESCC patients. Our results showed that patients who received ICI combined with chemotherapy/radiotherapy or CRT obtained an improved OS2 of 11.2 months compared to the previously reported outcomes of 2L chemotherapy (median OS2: 6.2–8.4 months) and 2L ICI alone (median OS2: 8.3–10.9 months) in patients with advanced ESCC [13, 15, 16, 17, 18].

Safety profiles in our study in the two groups were similar to the known data of ICI, chemotherapy, CRT, and target therapy, and no new safety signals were observed. Besides, the survival benefit of 2L ICI rechallenge did not come at the expense of significantly increased TRAEs (Grades 3–4 TRAEs: 43.1% vs. 37.9%). When it comes to irAEs, compared with the prior 1L ICI therapy, we did not observe an increase in the incidence of any grade of irAEs in the ICI rechallenge group (39.4% vs. 44.9%; *p* = 0.387), but a mild directional increase in the incidence of Grade 3–4 irAEs was observed (18.3% vs. 13.8%; *p* = 0.327) in this study. The spectrum of irAEs in patients before and after ICI rechallenge treatment was roughly similar, mainly including rash, thyroid dysfunction, pneumonia, hepatitis, etc. ICI rechallenge is a very important therapeutic strategy. Failure or intolerance to front‐line ICI is not a determining factor to deny subsequent immunotherapy. This also requires a comprehensive consideration of the survival benefits and quality of life of patients. It is reported that compared with disease progression, better clinical outcomes of ICI rechallenge were seen in patients who discontinued front‐line ICI treatment due to irAEs. Overall, these TRAEs were well controlled with symptomatic therapies and/or ICI treatment discontinuations with or without subsequent dose reduction. As a retrospective study, there may be considerable bias in the results due to incomplete documentation of TRAEs, which is one of the common drawbacks of such studies.

The study has some other limitations. First, selection bias and recall bias might exist in any retrospective study. Secondly, limited sample size reduced the statistical power. Thirdly, real‐world study results may be affected by some confounding factors, such as comorbidities. Fourth, the information loss of PD‐L1 is obvious, and the subgroup analysis results of survival benefits based on the PD‐L1 expression should be nevertheless interpreted with caution, and a prospective large‐sample clinical study is required for further confirmation. Fifth, further evaluation of biomarkers other than PD‐L1, EGFR, and Ki67 that might be associated with survival is warranted. However, despite these limitations, the present results provide valuable information for ICI rechallenge therapy in advanced ESCC patients at 2L setting after 1L ICI failure.

In conclusion, second‐line ICI rechallenge yielded an improved OS than non‐ICI rechallenge strategies for advanced or metastatic ESCC patients, with manageable safety. It might represent a potential standard option of 2L treatment for ESCC patients after 1L ICI failure. Further prospective study is warranted to confirm these results.

## Author Contributions

Conceptualization: Dedong Cao. Data curation: Wensi Zhao, Nan Zhao. Formal analysis: Wensi Zhao. Funding acquisition: Wensi Zhao. Investigation: all authors. Methodology: Wensi Zhao, Dedong Cao. Project administration: Dedong Cao. Supervision: Dedong Cao. Writing – original draft: Wensi Zhao. Writing – review and editing: Wensi Zhao, Dedong Cao.

## Ethics Statement

This study was approved by the Ethics Committee of Renmin Hospital of Wuhan University (Approval No.: 2023K‐K094).

## Consent

Written informed consent was obtained from all individual participants included in the study.

## Conflicts of Interest

The authors declare no conflicts of interest.

## Supporting information


**Table S1.** Patient baseline characteristics before and after propensity score matching. CPS, combined positive score; ECOG PS, Eastern Cooperative Oncology Group performance status; EGFR (i), epidermal growth factor receptor (inhibitor); ICI, immune checkpoint inhibitor; MT, mutated type; PD‐L1, programmed cell death‐ligand 1; WT, wild type.
**Table S2.** Univariate Cox regression analysis of prognostic factors for survival in patients of ICI rechallenge group. The bold font represents statistical significance at *p* < 0.05. CI, confidence interval; CPS, combined positive score; ECOG PS, Eastern Cooperative Oncology Group performance status; EGFR (i), epidermal growth factor receptor (inhibitor); HR, hazard ratio; ICI, immune checkpoint inhibitor; MT, mutated type; OS2, second‐line overall survival; PFS2, second‐line progression‐free survival; PD‐L1, programmed cell death‐ligand 1; TKI, tyrosine kinase inhibitor; VEGFi, vascular endothelial growth factor inhibitor; WT, wild type.
**Table S3.** Multivariate Cox regression analysis of prognostic factors for survival in patients of ICI crossline group. The bold font represents statistical significance at *p* < 0.05. CI, confidence interval; EGFR, epidermal growth factor receptor; HR, hazard ratio; ICI, immune checkpoint inhibitor; MT, mutated type; OS2, second‐line overall survival; PFS2, second‐line progression‐free survival; WT, wild type.
**Table S4.** Univariate and multivariate cox regression analysis of prognostic factors for overall survival in all patients of first‐line ICI‐based therapy. The bold font represents statistical significance at *p* < 0.05. CI, confidence interval; CPS, combined positive score; ECOG PS, Eastern Cooperative Oncology Group performance status; EGFR (i), epidermal growth factor receptor (inhibitor); HR, hazard ratio; ICI, immune checkpoint inhibitor; MT, mutated type; PD‐L1, programmed cell death‐ligand 1; WT, wild type.
**Figure S1.** Survival curves of patients after propensity score matching. (A, B) OS of second‐line treatment. (C, D) PFS of second‐line treatment. (E, F) OS of first‐line treatment. CI, confidence interval; HR, hazard ratio; ICI, immune checkpoint inhibitor; OS, overall survival; PFS, progression‐free survival.

## Data Availability

Data collected for the study, including individual participant data that underlie the results reported in this manuscript, after de identification, will be shared with investigators. Data collection and analyses were conducted in accordance with the principles of the Declaration of Helsinki.
