# Supplementary material for: Efficacy and Safety of Second‐Line Immune Checkpoint Inhibitor Rechallenge in Advanced or Metastatic Esophageal Squamous Cell Carcinoma: A Retrospective Study
Source: Thorac Cancer. 2025 Jul 9;16(13):e70131. doi: 10.1111/1759-7714.70131 (PMC12240725; doi:10.1111/1759-7714.70131)
Supplement: Supplementary file 1 — Table S1. Patient baseline characteristics before and after propensity score matching. CPS, combined positive score; ECOG PS, Eastern Cooperative Oncology Group performance status; EGFR (i), epidermal growth factor receptor (inhibitor); ICI, immune checkpoint inhibitor; MT, mutated type; PD‐L1, programmed cell death‐ligand 1; WT, wild type. Table S2. Univariate Cox regression analysis of prognostic factors for survival in patients of ICI rechallenge group. The bold font represents statistical significance at p < 0.05. CI, confidence interval; CPS, combined positive score; ECOG PS, Eastern Cooperative Oncology Group performance status; EGFR (i), epidermal growth factor receptor (inhibitor); HR, hazard ratio; ICI, immune checkpoint inhibitor; MT, mutated type; OS2, second‐line overall survival; PFS2, second‐line progression‐free survival; PD‐L1, programmed cell death‐ligand 1; TKI, tyrosine kinase inhibitor; VEGFi, vascular endothelial growth factor inhibitor; WT, wild type. Table S3. Multivariate Cox regression analysis of prognostic factors for survival in patients of ICI crossline group. The bold font represents statistical significance at p < 0.05. CI, confidence interval; EGFR, epidermal growth factor receptor; HR, hazard ratio; ICI, immune checkpoint inhibitor; MT, mutated type; OS2, second‐line overall survival; PFS2, second‐line progression‐free survival; WT, wild type. Table S4. Univariate and multivariate cox regression analysis of prognostic factors for overall survival in all patients of first‐line ICI‐based therapy. The bold font represents statistical significance at p < 0.05. CI, confidence interval; CPS, combined positive score; ECOG PS, Eastern Cooperative Oncology Group performance status; EGFR (i), epidermal growth factor receptor (inhibitor); HR, hazard ratio; ICI, immune checkpoint inhibitor; MT, mutated type; PD‐L1, programmed cell death‐ligand 1; WT, wild type. Figure S1. Survival curves of patients after propensity score matching. (A, B) OS of [file TCA-16-e70131-s001.docx]

**Supporting information**

| **Contents** |
| --- |
| **Table S1.** Patient baseline characteristics before and after propensity score matching. CPS, combined positive score; ECOG PS, Eastern Cooperative Oncology Group performance status; EGFR(i), epidermal growth factor receptor (inhibitor); ICI, immune checkpoint inhibitor; MT, mutated type; PD-L1, programmed cell death-ligand 1; WT, wild type. |
| **Table S2.** Univariate Cox regression analysis of prognostic factors for survival in patients of ICI rechallenge group. The bold font represents statistical significance at *P* < 0.05. CI: confidence interval; CPS, combined positive score; ECOG PS, Eastern Cooperative Oncology Group performance status; EGFR(i), epidermal growth factor receptor (inhibitor); HR: hazard ratio; ICI, immune checkpoint inhibitor; MT, mutated type; OS2, second-line overall survival; PFS2, second-line progression-free survival; PD-L1, programmed cell death-ligand 1; TKI, tyrosine kinase inhibitor; VEGFi, vascular endothelial growth factor inhibitor; WT, wild type. |
| **Table S3.** Multivariate Cox regression analysis of prognostic factors for survival in patients of ICI crossline group. The bold font represents statistical significance at *P* < 0.05. CI: confidence interval; EGFR, epidermal growth factor receptor; HR: hazard ratio; ICI, immune checkpoint inhibitor; MT, mutated type; OS2, second-line overall survival; PFS2, second-line progression-free survival; WT, wild type. |
| **Table S4.** Univariate and multivariate Cox regression analysis of prognostic factors for overall survival in all patients of first-line ICI-based therapy. The bold font represents statistical significance at *P* < 0.05. CI: confidence interval; CPS, combined positive score; ECOG PS, Eastern Cooperative Oncology Group performance status; EGFR(i), epidermal growth factor receptor (inhibitor); HR: hazard ratio; ICI, immune checkpoint inhibitor; MT, mutated type; PD-L1, programmed cell death-ligand 1; WT, wild type. |
| **Figure S1.** Survival curves of patients after propensity score matching. (A-B) OS of second-line treatment. (C-D) PFS of second-line treatment. (E-F) OS of first-line treatment. CI, confidence interval; HR, hazard ratio; ICI, immune checkpoint inhibitor; OS, overall survival; PFS, progression-free survival. |

**Table S1.** Patient baseline characteristics before and after propensity score matching.

| **Variables** | **Before Matching** | | | **After Matching** | | | |  |
| --- | --- | --- | --- | --- | --- | --- | --- | --- |
|  | **ICI cohort (N=109)** | **Non-ICI cohort (N=29)** | ***P*** |  | **ICI cohort (N=27)** | **Non-ICI cohort (N=27)** | ***P*** | |
| Age (year) |  |  | 0.176 |  |  |  | 1.000 | |
| ≥ 60 | 68 (62.4) | 22 (75.9) |  |  | 23 (85.2) | 22 (81.5) |  | |
| < 60 | 41 (37.6) | 7 (24.1) |  |  | 4 (14.8) | 5 (18.5) |  | |
| Sex |  |  | 1.000 |  |  |  | 1.000 | |
| Male | 94 (86.2) | 25 (86.2) |  |  | 23 (85.2) | 23 (85.2) |  | |
| Female | 15 (13.8) | 4 (13.8) |  |  | 4 (14.8) | 4 (14.8) |  | |
| ECOG PS |  |  | 1.000 |  |  |  | 1.000 | |
| 0-1 | 107 (98.2) | 29 (100.0) |  |  | 27 (100.0) | 27 (100.0) |  | |
| 2 | 2 (1.8) | 0 (0.0) |  |  | 0 (0.0) | 0 (0.0) |  | |
| Primary tumor stage |  |  | 1.000 |  |  |  | 1.000 | |
| I-II | 11 (10.1) | 3 (10.3) |  |  | 3 (11.1) | 3 (11.1) |  | |
| III-IV | 98 (89.9) | 26 (89.7) |  |  | 24 (88.9) | 24 (88.9) |  | |
| Primary tumor location |  |  | 0.088 |  |  |  | 0.770 | |
| Upper esophagus | 19 (17.4) | 2 (6.9) |  |  | 3 (11.1) | 2 (7.4) |  | |
| Middle esophagus | 55 (50.5) | 21 (72.4) |  |  | 21 (77.8) | 19 (70.4) |  | |
| Lower esophagus | 31 (28.4) | 4 (13.8) |  |  | 2 (7.4) | 4 (14.8) |  | |
| Unknown | 4 (3.7) | 2 (6.9) |  |  | 1 (3.7) | 2 (7.4) |  | |
| Distant metastasis |  |  | 0.731 |  |  |  | 1.000 | |
| Yes | 64 (58.7) | 16 (55.2) |  |  | 15 (55.6) | 15 (55.6) |  | |
| No | 45 (41.3) | 13 (44.8) |  |  | 12 (44.4) | 12 (44.4) |  | |
| Previous surgery |  |  | 0.075 |  |  |  | 0.248 | |
| Yes | 54 (49.5) | 9 (31.0) |  |  | 11 (40.7) | 7 (25.9) |  | |
| No | 55 (50.5) | 20 (69.0) |  |  | 16 (59.3) | 20 (74.1) |  | |
| Previous chemotherapy |  |  | 0.806 |  |  |  | 0.471 | |
| Yes | 105 (96.3) | 27 (93.1) |  |  | 27 (100.0) | 25 (92.6) |  | |
| No | 4 (3.7) | 2 (6.9) |  |  | 0 (0.0) | 2 (7.4) |  | |
| Previous radiotherapy |  |  | 0.538 |  |  |  | 0.091 | |
| Yes | 67 (61.5) | 16 (55.2) |  |  | 20 (74.1) | 14 (51.8) |  | |
| No | 42 (38.5) | 13 (44.8) |  |  | 7 (25.9) | 13 (48.2) |  | |
| Previous immunotherapy |  |  | 1.000 |  |  |  | 1.000 | |
| Yes | 109 (100.0) | 29 (100.0) |  |  | 27 (100.0) | 27 (100.0) |  | |
| No | 0 (0.0) | 0 (0.0) |  |  | 0 (0.0) | 0 (0.0) |  | |
| Previous EGFRi/VEGFi/TKI |  |  | 0.172 |  |  |  | 0.311 | |
| Yes | 21 (19.3) | 9 (31.0) |  |  | 4 (14.8) | 7 (25.9) |  | |
| No | 88 (80.7) | 20 (69.0) |  |  | 23 (85.2) | 20 (74.1) |  | |
| PD-L1 |  |  | 0.406 |  |  |  | 1.000 | |
| CPS < 1 | 2 (1.8) | 1 (3.4) |  |  | 1 (3.7) | 1 (3.7) |  | |
| 1 ≤ CPS < 10 | 5 (4.6) | 2 (6.9) |  |  | 2 (7.4) | 1 (3.7) |  | |
| CPS ≥ 10 | 7 (6.4) | 0 (0.0) |  |  | 0 (0.0) | 0 (0.0) |  | |
| Unknown | 95 (87.2) | 26 (89.7) |  |  | 24 (88.9) | 24 (88.9) |  | |
| EGFR status |  |  | 0.625 |  |  |  | 0.569 | |
| WT | 2 (1.8) | 0 (0.0) |  |  | 1 (3.7) | 0 (0.0) |  | |
| MT | 43 (39.5) | 9 (31.0) |  |  | 10 (37.0) | 8 (29.6) |  | |
| Unknown | 64 (58.7) | 20 (69.0) |  |  | 16 (59.3) | 19 (70.4) |  | |
| Ki67% expression |  |  | 0.611 |  |  |  | 0.467 | |
| ≤ 30 | 6 (5.5) | 3 (10.3) |  |  | 1 (3.7) | 3 (11.1) |  | |
| > 30 | 27 (24.8) | 6 (20.7) |  |  | 9 (33.3) | 6 (22.2) |  | |
| Unknown | 76 (69.7) | 20 (69.0) |  |  | 17 (63.0) | 18 (66.7) |  | |

Abbreviations: CPS, combined positive score; ECOG PS, Eastern Cooperative Oncology Group performance status; EGFR(i), epidermal growth factor receptor (inhibitor); ICI, immune checkpoint inhibitor; MT, mutated type; PD-L1, programmed cell death-ligand 1; WT, wild type.

**Table S2.** Univariate Cox regression analysis of prognostic factors for survival in patients of ICI rechallenge group.

| **Variables** | **N** | **OS2** | | **PFS2** | |
| --- | --- | --- | --- | --- | --- |
|  |  | **HR (95% CI)** | ***P*** | **HR (95% CI)** | ***P*** |
| Age (year) |  |  |  |  |  |
| < 60 | 41 | 1.00 (Reference) |  | 1.00 (Reference) |  |
| ≥ 60 | 68 | 0.98 (0.62 ~ 1.56) | 0.946 | 1.00 (0.66 ~ 1.51) | 0.989 |
| Sex |  |  |  |  |  |
| Female | 15 | 1.00 (Reference) |  | 1.00 (Reference) |  |
| Male | 94 | 1.70 (0.87 ~ 3.33) | 0.121 | 1.71 (0.92 ~ 3.17) | 0.088 |
| ECOG PS |  |  |  |  |  |
| 2 | 2 | 1.00 (Reference) |  | 1.00 (Reference) |  |
| 0-1 | 107 | 0.49 (0.12 ~ 2.02) | 0.323 | 0.61 (0.15 ~ 2.48) | 0.487 |
| Primary tumor stage |  |  |  |  |  |
| I-II | 11 | 1.00 (Reference) |  | 1.00 (Reference) |  |
| III-IV | 98 | 1.00 (0.48 ~ 2.08) | 0.990 | 1.07 (0.55 ~ 2.08) | 0.832 |
| Primary tumor location |  |  |  |  |  |
| Unknown | 4 | 1.00 (Reference) |  | 1.00 (Reference) |  |
| Upper esophagus | 19 | 0.59 (0.19 ~ 1.89) | 0.377 | 1.09 (0.36 ~ 3.29) | 0.875 |
| Middle esophagus | 55 | 0.63 (0.22 ~ 1.79) | 0.385 | 0.96 (0.34 ~ 2.68) | 0.938 |
| Lower esophagus | 31 | 0.74 (0.26 ~ 2.13) | 0.574 | 1.30 (0.45 ~ 3.69) | 0.627 |
| Distant metastasis |  |  |  |  |  |
| No | 45 | 1.00 (Reference) |  | 1.00 (Reference) |  |
| Yes | 64 | 0.98 (0.61 ~ 1.55) | 0.924 | 0.85 (0.56 ~ 1.27) | 0.422 |
| Prior surgery |  |  |  |  |  |
| No | 55 | 1.00 (Reference) |  | 1.00 (Reference) |  |
| Yes | 54 | 0.87 (0.56 ~ 1.35) | 0.534 | 0.71 (0.47 ~ 1.06) | 0.091 |
| Prior chemotherapy |  |  |  |  |  |
| No | 4 | 1.00 (Reference) |  | 1.00 (Reference) |  |
| Yes | 105 | 0.75 (0.18 ~ 3.08) | 0.686 | 0.86 (0.27 ~ 2.72) | 0.796 |
| Prior radiotherapy |  |  |  |  |  |
| No | 42 | 1.00 (Reference) |  | 1.00 (Reference) |  |
| Yes | 67 | 1.39 (0.87 ~ 2.21) | 0.170 | 1.06 (0.71 ~ 1.60) | 0.768 |
| Prior EGFRi/VEGFi/TKI |  |  |  |  |  |
| No | 88 | 1.00 (Reference) |  | 1.00 (Reference) |  |
| Yes | 21 | 0.90 (0.53 ~ 1.55) | 0.711 | 0.74 (0.44 ~ 1.25) | 0.263 |
| PD-L1 |  |  |  |  |  |
| Unknown | 95 | 1.00 (Reference) |  | 1.00 (Reference) |  |
| CPS < 1 | 2 | 0.00 (0.00 ~ Inf) | 0.996 | 0.80 (0.20 ~ 3.26) | 0.754 |
| 1 ≤ CPS < 10 | 5 | 0.24 (0.03 ~ 1.76) | 0.161 | 1.09 (0.34 ~ 3.49) | 0.879 |
| CPS ≥ 10 | 7 | 0.78 (0.34 ~ 1.81) | 0.570 | 0.50 (0.20 ~ 1.25) | 0.139 |
| EGFR status |  |  |  |  |  |
| Unknown | 64 | 1.00 (Reference) |  | 1.00 (Reference) |  |
| WT | 2 | 0.26 (0.04 ~ 1.91) | 0.185 | 0.84 (0.20 ~ 3.45) | 0.807 |
| MT | 43 | 0.55 (0.34 ~ 0.88) | **0.014** | 0.76 (0.50 ~ 1.15) | 0.195 |
| Ki67% expression |  |  |  |  |  |
| Unknown | 76 | 1.00 (Reference) |  | 1.00 (Reference) |  |
| ≤ 30 | 6 | 0.38 (0.09 ~ 1.55) | 0.176 | 0.70 (0.25 ~ 1.91) | 0.482 |
| > 30 | 27 | 0.76 (0.43 ~ 1.31) | 0.321 | 0.84 (0.53 ~ 1.34) | 0.468 |

The bold font represents statistical significance at *P* < 0.05.

Abbreviations: CI, confidence interval; CPS, combined positive score; ECOG PS, Eastern Cooperative Oncology Group performance status; EGFR(i), epidermal growth factor receptor (inhibitor); HR: hazard ratio; ICI, immune checkpoint inhibitor; MT, mutated type; OS2, second-line overall survival; PFS2, second-line progression-free survival; PD-L1, programmed cell death-ligand 1; TKI, tyrosine kinase inhibitor; VEGFi, vascular endothelial growth factor inhibitor; WT, wild type.

**Table S3.** Multivariate Cox regression analysis of prognostic factors for survival in patients of ICI crossline group.

| **Variables** | **N** | **OS2** | | **PFS2** | |
| --- | --- | --- | --- | --- | --- |
|  |  | **HR (95% CI)** | ***P*** | **HR (95% CI)** | ***P*** |
| Sex |  |  |  |  |  |
| Female | 15 | - |  | 1.00 (Reference) |  |
| Male | 94 | - | - | 1.61 (0.86 ~ 2.99) | 0.134 |
| Prior surgery |  |  |  |  |  |
| No | 55 | - |  | 1.00 (Reference) |  |
| Yes | 54 | - | - | 0.74 (0.50 ~ 1.11) | 0.152 |
| EGFR status |  |  |  |  |  |
| Unknown | 64 | 1.00 (Reference) |  | - |  |
| WT | 2 | 0.26 (0.04 ~ 1.91) | 0.185 | - | - |
| MT | 43 | 0.55 (0.34 ~ 0.88) | **0.014** | - | - |

The bold font represents statistical significance at *P* < 0.05.

Abbreviations: CI, confidence interval; EGFR, epidermal growth factor receptor; HR: hazard ratio; ICI, immune checkpoint inhibitor; MT, mutated type; OS2, second-line overall survival; PFS2, second-line progression-free survival; WT, wild type.

**Table S4.** Univariate and multivariate Cox regression analysis of prognostic factors for overall survival in all patients of first-line ICI-based therapy.

| **Variables** | **N** | **Univariate analysis** | | **Multivariate analysis** | |
| --- | --- | --- | --- | --- | --- |
|  |  | **HR (95% CI)** | ***P*** | **HR (95% CI)** | ***P*** |
| Age (year) |  |  |  |  |  |
| < 60 | 48 | 1.00 (Reference) |  | - |  |
| ≥ 60 | 90 | 0.74 (0.50 ~ 1.11) | 0.149 | - | - |
| Sex |  |  |  |  |  |
| Female | 19 | 1.00 (Reference) |  | - |  |
| Male | 119 | 1.32 (0.76 ~ 2.29) | 0.328 | - | - |
| ECOG PS |  |  |  |  |  |
| 2 | 2 | 1.00 (Reference) |  | 1.00 (Reference) |  |
| 0-1 | 136 | 0.30 (0.07 ~ 1.22) | 0.092 | 0.17 (0.04 ~ 0.74) | **0.018** |
| Primary tumor stage |  |  |  |  |  |
| I-II | 14 | 1.00 (Reference) |  | - |  |
| III-IV | 124 | 1.72 (0.86 ~ 3.44) | 0.122 | - | - |
| Primary tumor location |  |  |  |  |  |
| Unknown | 6 | 1.00 (Reference) |  | - |  |
| Upper esophagus | 21 | 0.61 (0.21 ~ 1.77) | 0.360 | - |  |
| Middle esophagus | 76 | 0.94 (0.36 ~ 2.42) | 0.890 | - |  |
| Lower esophagus | 35 | 1.15 (0.44 ~ 3.03) | 0.776 | - | - |
| Distant metastasis |  |  |  |  |  |
| No | 58 | 1.00 (Reference) |  | - |  |
| Yes | 80 | 1.38 (0.92 ~ 2.08) | 0.123 | - | - |
| Prior surgery |  |  |  |  |  |
| No | 89 | 1.00 (Reference) |  | - |  |
| Yes | 49 | 1.11 (0.74 ~ 1.66) | 0.609 | - | - |
| Prior chemotherapy |  |  |  |  |  |
| No | 100 | 1.00 (Reference) |  | - |  |
| Yes | 38 | 1.14 (0.75 ~ 1.74) | 0.535 | - | - |
| Prior radiotherapy |  |  |  |  |  |
| No | 116 | 1.00 (Reference) |  | - |  |
| Yes | 22 | 1.31 (0.80 ~ 2.15) | 0.276 | - | - |
| Prior immunotherapy |  |  |  |  |  |
| No | 130 | 1.00 (Reference) |  | - |  |
| Yes | 8 | 0.98 (0.40 ~ 2.42) | 0.969 | - | - |
| Prior EGFRi |  |  |  |  |  |
| No | 133 | 1.00 (Reference) |  | - |  |
| Yes | 5 | 1.40 (0.57 ~ 3.46) | 0.462 | - | - |
| PD-L1 |  |  |  |  |  |
| Unknown | 121 | 1.00 (Reference) |  | 1.00 (Reference) |  |
| CPS < 1 | 3 | 0.30 (0.04 ~ 2.15) | 0.230 | 0.32 (0.04 ~ 2.45) | 0.275 |
| 1≤ CPS < 10 | 7 | 0.09 (0.01 ~ 0.62) | **0.015** | 0.07 (0.01 ~ 0.57) | **0.013** |
| CPS ≥ 10 | 7 | 0.57 (0.23 ~ 1.42) | 0.225 | 0.67 (0.26 ~ 1.70) | 0.395 |
| EGFR status |  |  |  |  |  |
| Unknown | 84 | 1.00 (Reference) |  | 1.00 (Reference) |  |
| WT | 2 | 0.34 (0.05 ~ 2.49) | 0.289 | 5.12 (0.64 ~ 40.79) | 0.123 |
| MT | 52 | 0.57 (0.38 ~ 0.87) | **0.010** | 0.63 (0.40 ~ 0.98) | **0.038** |
| Ki67% expression |  |  |  |  |  |
| Unknown | 96 | 1.00 (Reference) |  | 1.00 (Reference) |  |
| ≤ 30 | 9 | 0.20 (0.05 ~ 0.81) | **0.025** | 0.17 (0.04 ~ 0.76) | **0.020** |
| > 30 | 33 | 0.80 (0.50 ~ 1.29) | 0.367 | 0.79 (0.47 ~ 1.30) | 0.349 |
| 2L ICI rechallenge |  |  |  |  |  |
| No | 29 | 1.00 (Reference) |  | 1.00 (Reference) |  |
| Yes | 109 | 0.56 (0.35 ~ 0.89) | **0.015** | 0.37 (0.22 ~ 0.61) | **<0.001** |

The bold font represents statistical significance at *P* < 0.05.

Abbreviations: CI, confidence interval; CPS, combined positive score; ECOG PS, Eastern Cooperative Oncology Group performance status; EGFR(i), epidermal growth factor receptor (inhibitor); HR: hazard ratio; ICI, immune checkpoint inhibitor; MT, mutated type; PD-L1, programmed cell death-ligand 1; WT, wild type.

**Fig. S1.** Survival curves of patients after propensity score matching. (A-B) OS of second-line treatment. (C-D) PFS of second-line treatment. (E-F) OS of first-line treatment. Abbreviations: CI, confidence interval; HR, hazard ratio; ICI, immune checkpoint inhibitor; OS, overall survival; PFS, progression-free survival.
